# Biopolymer-Waste Fiber Reinforcement for Earthen Materials: Capillary, Mechanical, Impact, and Abrasion Performance

**DOI:** 10.3390/polym12081819

**Published:** 2020-08-13

**Authors:** Héctor Gonzalez-Calderon, Gerardo Araya-Letelier, Sabine Kunze, Claudia Burbano-Garcia, Úrsula Reidel, Cristián Sandoval, Rodrigo Astroza, Fernando Bas

**Affiliations:** 1 Facultad de Agronomía e Ingeniería Forestal, Pontificia Universidad Católica de Chile, Casilla 306, Correo 22, Santiago 7820436, Chile; hmgonzalez@uc.cl (H.G.-C.); fbas@uc.cl (F.B.); 2 Escuela de Construcción Civil, Pontificia Universidad Católica de Chile, Casilla 306, Correo 22, Santiago 7820436, Chile; sakunze@uc.cl (S.K.); cpburbano@uc.cl (C.B.-G.); 3 Grupo Materiales Compuestos (GMC), Escuela de Ingeniería de Materiales, Universidad del Valle, Cali 760034, Colombia; 4 Target Market Concrete, Sika S.A. Chile, Santiago 8941077, Chile; reidel.ursula@cl.sika.com; 5 Departamento de Ingeniería Estructural y Geotécnica y Escuela de Arquitectura, Pontificia Universidad Católica de Chile, Casilla 306, Correo 22, Santiago 7820436, Chile; csandoval@ing.puc.cl; 6 Universidad de los Andes, Chile, Facultad de Ingeniería y Ciencias Aplicadas, Santiago 7620001, Chile; rastroza@miuandes.cl

**Keywords:** biopolymer fiber, waste chicken feathers, fiber-reinforced earthen mixes, capillarity, impact strength, abrasion resistance

## Abstract

The poultry industry, highly prevalent worldwide, generates approximately 7.7 × 10^6^ metric tons of chicken feathers (CFs), which become a major environmental challenge due to their disposal when considered waste or due to their energy transformation consumption when considered by-products. CFs are mainly composed of keratin (approximately 90%), which is one of the most important biopolymers whose inherent characteristics make CFs suitable as biopolymer fibers (BPFs). This paper first assesses the morphological and chemical characteristics of these BPFs, through scanning electron microscopy and energy dispersive X-ray spectroscopy, and then evaluates the waste valorization of these BPFs as a sustainable alternative for fiber-reinforcement of earthen mixes intended for earthen construction, such as adobe masonry, rammed earth, and earthen plasters. In particular, four earthen mixes with increasing doses of BPFs (i.e., 0%, 0.25%, 0.5%, and 1% of BPFs by weight of soil) were developed to evaluate the impact of BPF-reinforcement on the capillary, mechanical, impact, and abrasion performance of these earthen mixes. The addition of BPFs did not significantly affect the mechanical performance of earthen mixes, and their incorporation had a statistically significant positive effect on the impact performance and abrasion resistance of earthen mixes as the BPF dose increased. On the other hand, the addition of BPFs increased the capillary water absorption rate, possibly due to a detected increment in porosity, which might reduce the durability of water-exposed BPF-reinforced earthen mixes, but a statistically significant increment only occurred when the highest BPF dose was used (1%).

## 1. Introduction

In both developed and developing countries, the poultry industry is characterized by large and increasing production volumes, as stated by the Food and Agriculture Organization of the United Nations, which revealed that the chicken production reached approximately 95 × 10^6^ metric tons in 2018 and 99 × 10^6^ metric tons in 2019 [1,2]. Currently, the USA, China, and Brazil are the worldwide leading countries in chicken production, reaching volumes of 19.3 × 10^6^, 13.8 × 10^6^, and 13.6 × 10^6^ metric tons, respectively, in 2019 [1]. The European Union follows them, with a production of 12.6 × 10^6^ metric tons [3]. This large and increasing worldwide chicken production generates an enormous amount of chicken feathers (CFs), since it is estimated that CFs represent 6% of a chicken’s weight [4,5]. Consequently, approximately 5.9 × 10^6^ metric tons of CFs were produced last year considering the corresponding chicken production of 2019.

These CFs, either considered as waste or as by-product derived from the poultry industry, have a high environmental impact worldwide due to their large and increasing volume [4,6,7]. When considered waste, these CFs are usually disposed of in landfills and/or treated in incineration plants [5,8,9] and their inadequate disposal generates environmental impacts, as well as the transmission of diseases [5]. Alternatively, when considered a by-product, these CFs are incorporated into low-quality feed supplements for animals [5,10,11], but prior to their incorporation, these CFs are subjected to elevated temperature and pressure processes (e.g., milling, boiling, and alkaline hydrolysis), which are expensive and require high demands of water and energy, leading to major environmental impacts worldwide [10,12,13].

Considering both the large production and significant environmental problems related to CFs, the United States Department of Agriculture patented a method to separate the three different structural levels of CFs into fiber pulp (from rachis and used to produce elements such as paper and filters) and fibers (from barbs and barbules and used as fiber-reinforcement for different materials) [14]. The latter patented method greatly eased the production of fibers from CFs.

**Figure 1:**
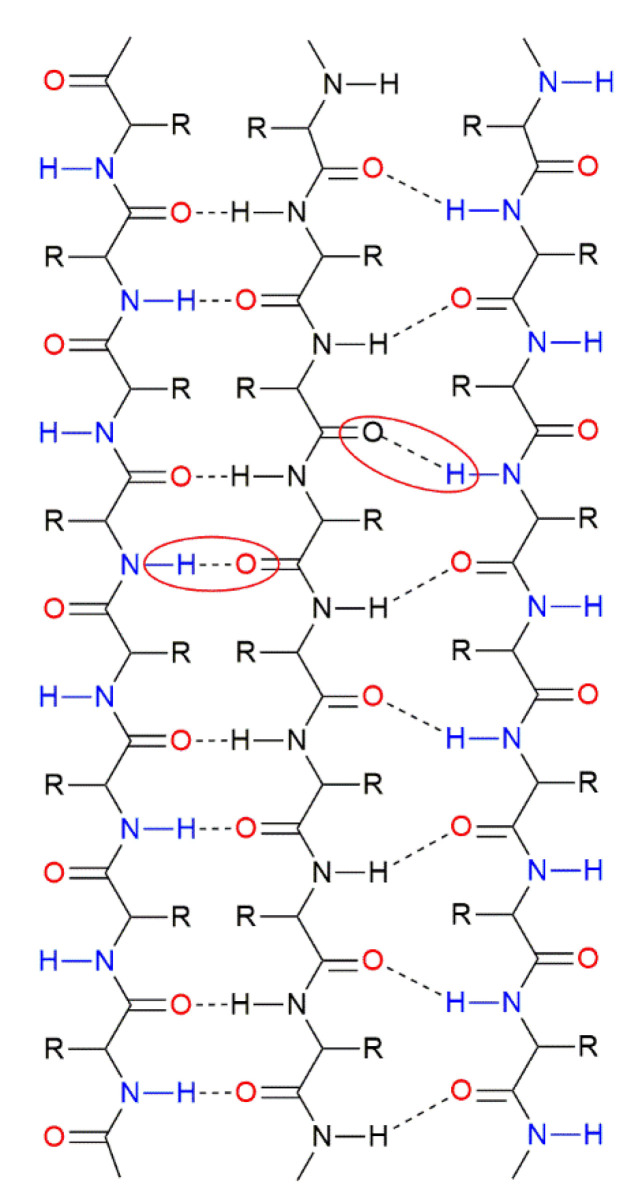
Pleated sheet structure of β-keratin.

These CFs can be considered keratin-based elements, since their composition is mainly based on this biopolymer (keratin) [10]. The latter is a relevant factor, since the development of keratin-based materials might have a large impact on the area of green construction materials due to characteristics such as biodegradability, biocompatibility, the mechanical performance, and the natural abundance of these keratin-based materials [15]. As CFs are a massive waste/by-product generated by the poultry industry worldwide [4] and their composition is largely based on keratin (approximately 90%), these CFs have attracted significant attention as fiber reinforcements for composite materials (e.g., [4,6,10,11,16,17,18,19,20,21]). These keratin-based feathers are small proteins with a primary structure based on amino acids and a molecular weight that ranges between 10 and 22 kDa [2,4]. Specifically, the chemical composition of these CFs presents cysteine (7.8 mol %), glycine (13.7 mol %), proline (9.8 mol %), serine (14.1 mol %), hydrophobic residues, and β-pleated sheets [2,3,4,7]. The type of keratin found in CFs (β-keratin) [22,23,24,25] exhibits a pleated sheet structure, as shown in Figure 1, consisting of β-strands connected laterally (either parallel or non-parallel) through hydrogen intermolecular bonds (see the red circle in Figure 1) [22]. This pleated sheet structure is stable due to two main factors: (i) The hydrogen bond between β-strands allows the generation of a sheet and (ii) the planar peptide bond induces a folded sheet [22]. Moreover, the peptide skeleton exhibits several functional groups, such as disulfide (–S–S), amine (–NH_2_), and carboxylic acid (–COOH) [15]. Therefore, keratin-based CFs can be defined as biopolymers and the fibers obtained from CFs are defined as biopolymer fibers (BPFs) in this study.

These BPFs have been morphologically, physically, and chemically characterized in several studies, whose results have highlighted their very low density (approximately 0.8 g/cm^3^) and good thermal and sound-damping performance, among other properties [9,26]. In addition, these BPFs have been studied as fiber-reinforcement for generic composite materials (e.g., [6,8,19,24,27,28,29,30]) and also as fiber-reinforcement for cement-based and plastic-based construction composite materials (e.g., [7,11,19,22,27,28]). Nevertheless, the use of these BPFs as fiber-reinforcement for non-synthetic construction materials has been included in very few studies, which have mainly been focused on the use of BPFs as reinforcement in soil remediation applications [13,29,30]. To the best of the authors’ knowledge, although a large fraction of the human population still lives in earthen-based dwellings [31] that exhibit limitations, such as a low impact and abrasion resistance, among others [32,33,34], the recent paper by Araya-Letelier et al. [35] is the only study addressing the use of these BPFs as fiber-reinforcement for earthen materials for construction applications. Specifically, the study by Araya-Letelier et al. [35] evaluated the effects of increasing BPF doses on the bulk density, compressive and flexural strength, drying shrinkage cracking, and water erosion performance of earthen mixes. The study found that a 1% BPF dose (in weight of BPFs to weight of oven-dry soil) did not statistically affect both the compressive and flexural strengths, but positively reduced the bulk density, drying shrinkage cracking, and water erosion of the fiber-reinforced mixes with respect to a plain (unreinforced) mix. Although the study by Araya-Letelier et al. [35] presents initial positive characteristics of this novel application of BPF-reinforcement of earthen materials, there are still several other durability and fracture performance properties that should be evaluated to further characterize the behavior of this BPF-reinforced earthen material for construction applications. Therefore, the originality of this study is the design and experimental assessment of the durability and fracture performance properties of BPF-reinforced earthen mixes that fulfill research gaps derived from the initial study by Araya-Letelier et al. [35] to further contribute to a necessary broader characterization of BPF-reinforced earthen materials for construction applications, such as rammed earth, adobe masonry, and earthen plasters.

The goals of this paper are to assess the influence of increasing doses of BPFs on earthen mixes (also considering an unreinforced earthen mix used as a control mix), in order to produce a broad experimental evaluation of the durability, mechanical, and fracture properties of this new composite material for construction applications. Specifically, the following properties of earthen mixes are assessed: (i) Capillarity; (ii) compressive and flexural strength; (iii) impact strength (both at first crack and at collapse); and (iv) dry abrasion resistance.

## 2. Materials and Methods

### 2.1. Material Characterization

#### 2.1.1. Clayey Soil

**Figure 2:**
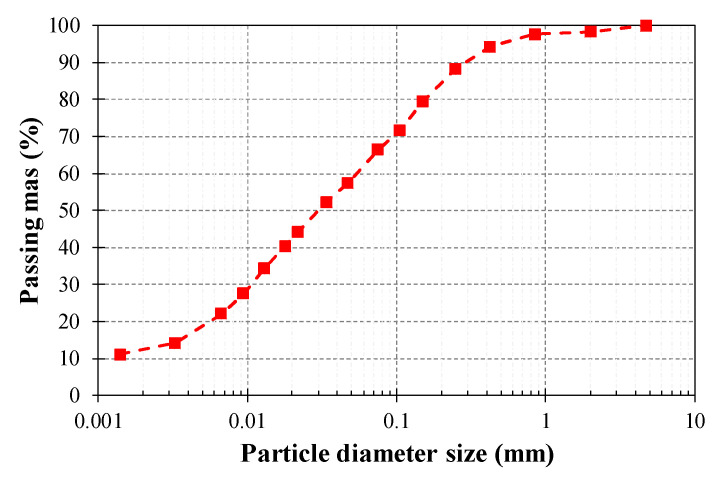
Particle diameter size distribution curve of clayey soil used in this research.

In order to manufacture the earthen mixes of this study, the same clayey soil used by Araya-Letelier et al. [35] was selected, whose particle diameter size distribution was determined by sieving and hydrometer analyses, following the standards ASTM D6913/D6913-17 [36] and ASTM D7928-17 [37], respectively. The resulting soil gradation curve is provided in Figure 2. The particle diameter size distribution was complemented by the determination of the Atterberg limits of the soil, following the standard ASTM D4318-17e1 [38], and the results are also shown in Figure 2. Considering the obtained particle diameter size distribution, as well as the Atterberg limits, this soil was classified as a low plasticity clay (CL), following the standard ASTM D2487-17e1 [39].

#### 2.1.2. BPFs

**Figure 3:**
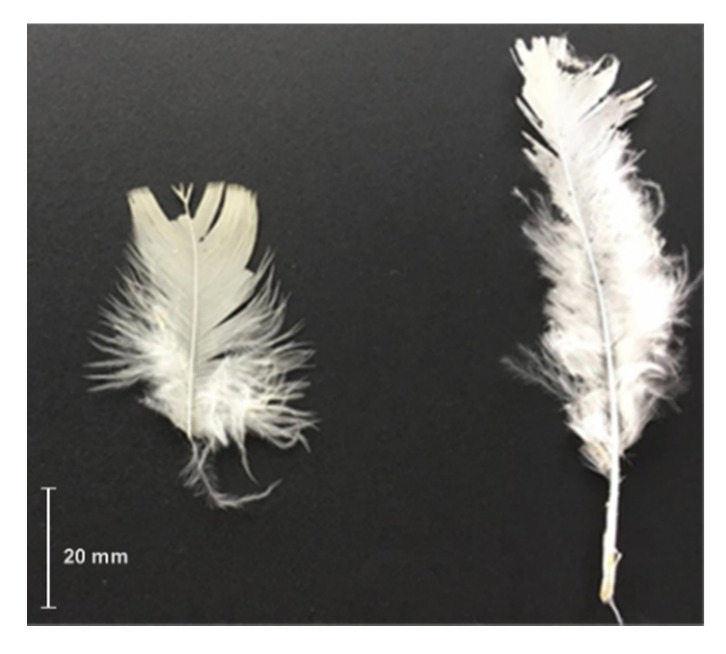
Chicken feathers (CFs) as received from a Chilean poultry company.

**Figure 4:**
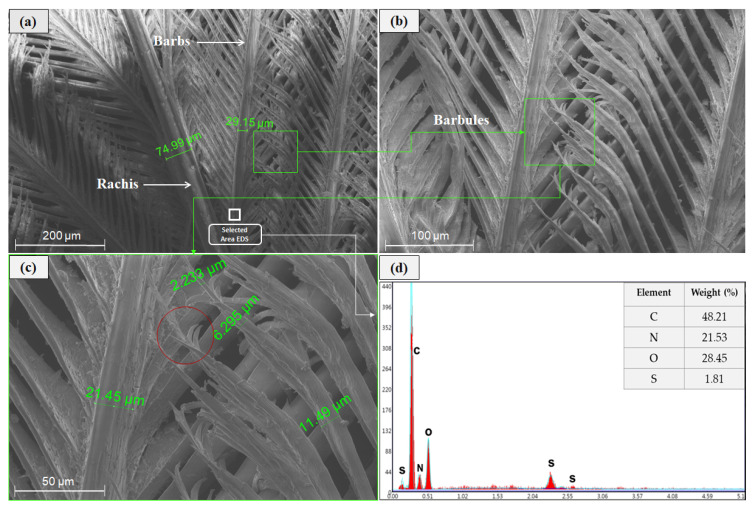
Scanning electron microscopy (SEM) of CFs: (**a**) Overall structure of CFs and selected area for energy dispersive X-ray spectroscopy (EDS) analysis; (**b**,**c**) overall structure of barbs and barbules; and (**d**) results of EDS analysis.

This paper used CFs obtained from a Chilean poultry company (see Figure 3), which were cleaned as suggested by Dalhat et al. [40] and processed to obtain BPFs. As mentioned previously, CFs exhibit three structural levels, which, in a decreasing dimension order, are rachis, barbs, and barbules, as shown in Figure 4. BPFs were obtained from these CFs by separating barbs and barbules from rachises and these rachises were disposed of, since they are stiff, thick, and not suitable as fibers [41].

As several studies have addressed these BPFs, this paper presents some of the main physical and mechanical characteristics of these BPFs obtained from previous studies, which are complemented with morphological characteristics (length, diameter, and aspect ratio) obtained using microscopy analysis over a sample of 50 BPFs, as well as the water absorption of these BPFs obtained by the paper towel method [42], which has been used in previous studies addressing the water absorption of other natural fibers (e.g., [43,44]). A summary of the main characteristics of these BPFs is presented in Table 1.

As can be seen in Figure 4a to Figure 4c, scanning electron microscopy (SEM) analysis was also implemented to study the microstructure of CFs and it clearly identified how the rachises (primary structural level of CFs) support the bars (secondary structural level) and, subsequently, the barbs support the barbules (third structural level). It can be seen that the rachis diameter is approximately 2.5 times the barb diameter, which is consistent with previous studies that defined the rachises as stiff and thick and not suitable as fiber-reinforcement. In comparison, these studies determined that the structure formed by barbs and barbules can be used as a fiber due to its high aspect ratio, flexibility, and strength [41]. It is worth noting that barbules and barbs (see Figure 4c) present a diameter ratio of approximately 1:2 at the barbules’ base (connection point between barbs and barbules), but barbule diameters are monotonically reduced from the base to the tip of the barbules. Moreover, as shown by the red circle in Figure 4c, barbules exhibit some ramifications at their tips that might have several implications: (i) They can further increase the mechanical bonding between the earthen matrix and these BPFs, and (ii) they can also increase the capillary water absorption since, as one barbule is further divided into three (or more) smaller filaments, the water travelling along a barbule can have three (or more) alternative directions to further continue the capillary diffusion through the earthen matrix.

Additionally, to chemically characterize these BPFs, this study also implemented energy dispersive X-ray spectroscopy (EDS) in the selected barb area shown in Figure 4a, whose results are presented in Figure 4d. The resulting weight composition showed the following chemical elements: C (48.21%), O (28.45%), N (21.53%), and S (1.81%). This is consistent with previous studies (e.g., [40]). The latter chemical composition can be related to the principal functional groups present in the biopolymer keratin, which is the main component of these BPFs. Lastly, the chemical elements carbon and oxygen can be related to the functional carboxylic acid groups (–COOH), the presence of nitrogen can be related to the amino groups (–NH_2_), and the sulfur can be related to the presence of disulfide groups (–S–S) [16,26].

### 2.2. Earthen Mixes and Specimen Preparation

Four earthen mixes with increasing doses of BPFs (0%, 0.25%, 0.5%, and 1% of BPFs by weight of oven-dry clayey soil, where the 0% dose corresponds to a plain earthen mix used as a control mix) were developed to evaluate the impact of BPF-reinforcement on their capillary, mechanical, impact, and abrasion performance.

The standard SENCICO E.80 [46] was implemented to produce the earthen mixes of this paper. The manufacturing process started by oven-drying the clayey soil for 24 h at 100 °C to eliminate any moisture (until reaching a constant mass), and the oven-dry soil was then kept covered and sealed for another 24 h to cool it down and avoid water absorption. Similarly, the latter process was also applied to BPFs. Following this, oven-dry BPFs were gradually added (in one, two, and four steps for 0.25%, 0.5%, and 1% doses of BPFs, respectively) to the oven-dry clayey soil, in fiber-reinforced mixes, and manual dry-mix was implemented to promote a uniform distribution of these BPFs that avoided the generation of fiber clusters. This intensive manual mixing process was applied for at least 40 min in the case of fiber-reinforced mixes in laboratory environmental conditions (22 °C and 45% relative humidity). Subsequently, water was gradually added (the total amount of water per mix was divided into three parts) and manual mixing was implemented until uniform mixes were produced, which required at least 30 min. It is worth mentioning that a weight of water to weight of oven-dry soil ratio (W/S) of 25% was used in this study, which was obtained from the cigar test (a simple field test employed to evaluate the binding force of a clayey soil used for earthen construction [47]). Additionally, extra water was added to fiber-reinforced mixes to offset the water absorption of the BPFs (e.g., 0.85 kg of extra water was added for each kg of oven-dry BPFs to offset the 85% water absorption exhibited by BPFs).

**Figure 5:**
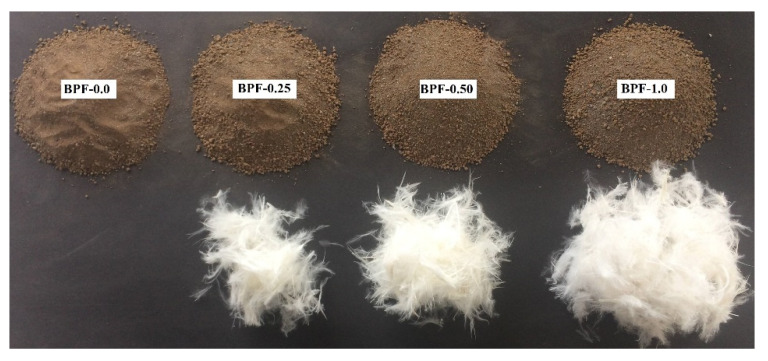
Proportions of oven-dry BPFs and oven-dry clayey soil of each earthen mix.

After the mixing process, each earthen mix was covered and sealed with a plastic film for three hours to keep the mix humidity constant and to promote a uniform water distribution within each mix. An identification (ID) code was given to each earthen mix and these ID codes reveal the weight percentage of oven-dry BPFs to oven-dry clayey soil. Table 2 presents the ID codes, as well as the material proportions of each mix, and Figure 5 graphically presents the proportions between oven-dry BPFs and oven-dry clayey soil for each earthen mix.

As detailed in Table 3, four different series of specimens were manufactured in this study for each of the earthen mixes presented in Table 2. Prismatic, beam, and cube specimens were cast over melamine wood molds, whereas RILEM beam specimens were cast over metallic molds, but both melamine and metallic molds were previously wetted to reduce the adherence between specimens and molds. Specimen compaction was manually executed, which has been commonly used in previous studies addressing earthen mixes (e.g., [34,48]). First, the edges of the specimens were filled with earthen mixes and manual compaction was then implemented using a tamper and considering layers of approximately 20 mm, in order to minimize the void content of mixes. RILEM beam and cube specimens were demolded 48 h after casting, whilst prismatic and beam specimens were demolded right after casting. Since the mechanical strength of earthen materials is highly affected by the moisture content, this study implemented a curing process where all specimens were conserved at laboratory environmental conditions (22 °C and 45% relative humidity) and rotated 90° to the adjacent side every seven days to promote a uniform curing process. This curing process was applied for 28 days before testing, when all of the specimens exhibited a constant mass (equilibrium between the moisture content of the specimens and the laboratory conditions).

### 2.3. Test Methods

#### 2.3.1. Capillarity

Due to their clay-based matrix, earthen construction materials naturally present high water absorption and even swelling, which can affect their durability. As the incorporation of fibers has been shown to increase the capillary water absorption in other materials, such as concrete [49], and since BPFs are characterized by a high absorption, this study addresses the effect of these BPFs on the capillary water absorption performance of earthen mixes.

The standard ASTM C1585-13 [50], aimed at evaluating the rate of capillary water absorption of concrete, was adapted in this study to evaluate the effect of BPFs on the water absorption rate due to the capillarity of earthen mixes. Prismatic specimens were oven-dried at 60 °C for 24 h (i.e., until reaching a constant mass), after which acrylic waterproof coatings were applied to each of the four lateral faces of each parallelepiped, which were left to dry out for three hours. Next, the specimens were again oven-dried at 60 °C for another 24 h. Subsequently, uncoated specimen faces were placed over fully saturated foams inside plastic boxes, in order to promote capillary water absorption without damaging the specimens due to direct water exposure. Then, these boxes were covered and sealed to prevent humidity variations. Finally, mass measurements were taken at time intervals of 0, 60 s, 5 min, 10 min, 20 min, 30 min, 60 min, 1 day, 2 days, 3 days, 6 days, 7 days, and 9 days after the beginning of the test to calculate the capillary water absorption rate using Equation (1).

(1) $$I=\frac{∆m}{a\cdot d},$$

where I represents the capillary water absorption rate, ∆m is the mass variation between two consecutive measurements, a is the cross section exposed to water absorption, and d is the water density.

The initial water absorption was determined in the time interval from 0 s to 6 h and the secondary water absorption was determined in the time interval between 1 day and 9 days. Values of *I* were plotted as a function of the time square root for each earthen mix.

It is worth noting that increments in capillary water absorption might be a consequence of the additional porosity generated by the addition of fibers to the matrix, as reported by previous studies [51,52,53]. Consequently, this study also calculated the porosity of each earthen mix, in order to understand how their microstructures affect their capillary water absorption values. Although the porosity can be estimated using nondestructive tests, such as tomography, porosity values can also be estimated based on the bulk density values of earthen mixes and the specific weight of solids of the clayey soil, as explained by Osman [54] and shown in Equation (2).

(2) $$P=\left(1-\frac{D}{W_{s}}\right)\cdot 100,$$

where P is the porosity (in percentage), D represents the bulk density, and $W_{s}$ is the specific weight of solids of the clayey soil (2469 kg/m^3^, as shown in Figure 2).

Bulk density values were simply calculated as the mass of solids, determined at the point of equilibrium moisture in laboratory conditions (22 °C and 45% relative humidity), divided by the total volume (including both soil and pore volumes). The total volume was determined based on 12 measurements (four for each width, height, and length) per prismatic specimen using a caliper (±0.02 mm precision), as suggested by Gandia et al. [55].

#### 2.3.2. Compressive and Flexural Strength

The standards ASTM C348-20 [56] and ASTM C349-18 [57] were adopted to evaluate the flexural and compressive strength, respectively, of each earthen mix, at the age of 28 days, using RILEM beam specimens. As pointed out in the standard ASTM C348 [56], each RILEM beam specimen was tested under flexion and then, after flexural fracture, two pieces were obtained from each specimen and these pieces were tested under compression following the standard ASTM C349-18 [57]. Consequently, six and twelve values were obtained for the estimation of the flexural strength and compressive strength, respectively, as shown in Table 3. For each earthen mix, the average (AV), standard deviation (SD), and coefficient of variation (COV) of flexural and compressive strength values were estimated at 28 days after casting (until reaching a constant mass).

It is worth mentioning that although the paper by Araya-Letelier et al. [35] evaluated the compressive and flexural strength of equivalent earthen mixes, the present study also assessed the mechanical properties. This was conducted to validate the manufacturing process employed to develop the new specimens in this study, by comparing the data with the compressive and flexural strength results of Araya-Letelier et al. [35] and, in order to obtain reliable results of the newly evaluated properties of capillary water absorption, impact strength and abrasion resistance of the BPF-reinforced earthen mixes addressed in this study.

#### 2.3.3. Impact Strength

**Figure 6:**
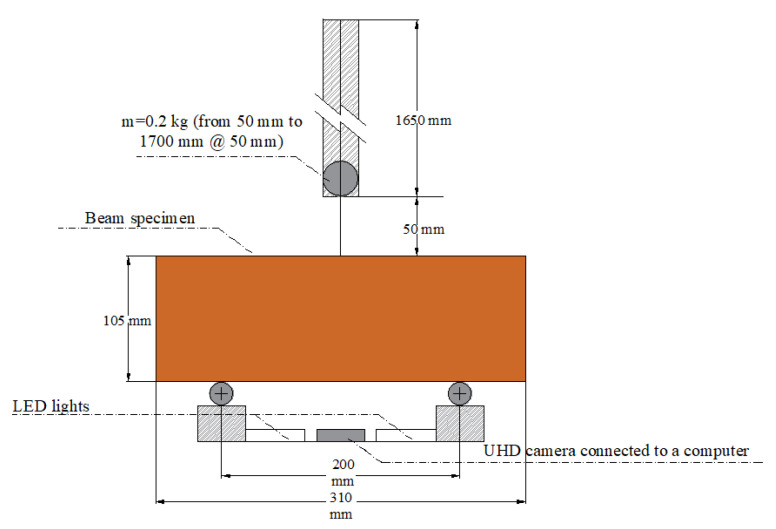
Impact strength test setup.

As mentioned by previous studies (e.g., [58,59,60,61]), the fracture toughness of composite materials, including earthen mixes, can be modified due to fiber-reinforcement and the impact strength test has been suggested as a way of measuring this damage-absorption capacity. This research implemented a setup consisting of beam specimens supported at two points by a metallic base (20 cm distance between supports) and a metallic projectile was thrown at the midspan of the beam specimens at increasing heights (see Figure 6). It is worth mentioning that two LED lights, as well as an ultra-high-definition (UHD) camera (with a protective case) connected to a computer, were placed at the bottom of the specimens to assist with the evaluation of the damage progress (especially with the identification of the generation of the first crack of each beam specimen), as shown in Figure 6. In the cases where beam specimens resisted the maximum drop height of the metallic projectile, subsequent drops were executed at the maximum height until collapse of the specimens was reached. Considering the mas of the metallic projectile (0.2 kg), the gravitational acceleration constant (9.81 m/s^2^) and the varying height from which the projectile was thrown (from 50 to 1700 mm, in 50 mm increments), the impact energy applied per blow ranged from 0.098 to 3.332 J for drop heights of 50 to 1700 mm, respectively.

Since the drops needed to generate the first crack and the collapse of each beam specimen were counted, then, the accumulated impact energy AV, SD, and COV values were estimated at 28 days after casting.

#### 2.3.4. Dry Abrasion Resistance

The standard XP P132-901 [62] was implemented to evaluate the dry abrasion resistance of the earthen mixes, considering six cube specimens per mix. This standard measures the mass of material removed after brushing the specimen during 60 cycles (each cycle corresponds to an application of the brush back and forth), where each cycle lasts for one second, and the procedure was performed using a metallic brush whose weight was 3 kg. As suggested by Giroudon et al. [63], the brushing process was implemented along the entire length of the cubes and at least one half of the brush area was permanently in contact with the cubes to avoid cantilever loading that could overstress the edge of the specimens. Numerically, the dry abrasion resistance was estimated for each specimen using the dry abrasion coefficient shown in Equation (3).

(3) $$C_{a}=\frac{S}{m_{0}-m_{1}},$$

where $C_{a}$ is the dry abrasion coefficient (expressed in cm^2^/g), S is the brushed area, $m_{0}$ is the mass before brushing, and $m_{1}$ is the mass after brushing. For each earthen mix, the AV, SD, and COV of dry abrasion coefficient values were estimated at 28 days after casting.

### 2.4. Analysis of Variance of Results

Since the analysis of variance (ANOVA) has been extensively used in previous investigations addressing the statistical significance of incorporating different reinforcements into earthen mixes (e.g., [48,64]), this study also implemented the ANOVA test to statistically evaluate the significance of the incorporation of BPFs in terms of the capillary, mechanical, impact, and abrasion performance of earthen mixes.

Firstly, one-way ANOVA tests were implemented in order to assess the statistical significance of the difference among the average performance of the study groups (i.e., BPF-0.0, BPF-0.25, BPF-0.50, and BPF-1.0) under a specific experimental test (e.g., impact strength). The one-way ANOVA tests assessed the null hypothesis (H_0_), which was that the average performance values of all earthen mixes were equivalent (i.e., BPFs did not modify the average performance values under study), against the alternative hypothesis (H_A_), which was that at least one average performance value was different. The 5% significance level considered in this study was consistent with previous studies (e.g., [48,65]).

If the null hypothesis was rejected, pair-wise single-factor ANOVA tests were applied to determine if, individually, each BPF-reinforced earthen mix performed, on average, differently when compared to the plain (unreinforced) earthen mix under a given experimental test.

Initially, the implementation of the ANOVA tests requires the calculation of a critical F value (F_crit_), defined as the corresponding value of an F-distribution that captures a specific area (e.g., 1%, 5%, or 10% of the total area) under the right tail of the distribution. The F_crit_ value depends on (i) the significance level (5%), (ii) the number of study groups (e.g., four earthen mixes in this paper in the case of one-way ANOVA tests), and (iii) the number of values observed for each study group (e.g., six specimens for each earthen mix in the case of the impact strength test).

Subsequently, a statistical f value (f_st_), defined as the ratio of two mean squares, which are variances that account for the degrees of freedom used to estimate these values, was calculated. The numerator is a mean square value that accounts for the variance between the study groups, whereas the denominator is a mean square value that accounts for the variance within each study group. Values of f_st_ are calculated for each ANOVA test and compared to F_crit_. Then, if f_st_ is less than F_crit_, it implies that the differences among the average performance values of the four earthen mixes under a given test are not statistically significant (one-way ANOVA test) or that the differences among the average performance values of two specific earthen mixes are not statistically significant (pair-wise ANOVA test).

The basic foundation of the ANOVA test is that differences between sample averages can be explained by two possible reasons: (i) Sample averages originate from different populations (in this study, they correspond to the incorporation of BPFs), and (ii) sample averages originate from the same population and, consequently, differences are the result of chance and/or sampling error. The implementation of ANOVA tests demands the calculation of sums of squares (SS), means of squares (MS), and degrees of freedom (DF) for both the treatment and the error, and these calculations are provided for each ANOVA test implemented in this study.

Finally, this paper reports the *p*-value, defined as the probability of finding the observed results, or even more extreme results when the null hypothesis is true, of each ANOVA test performed. Further information about the implementation of the ANOVA tests can be found in [66].

## 3. Experimental Results and Analyses

### 3.1. Capillarity

**Figure 7:**
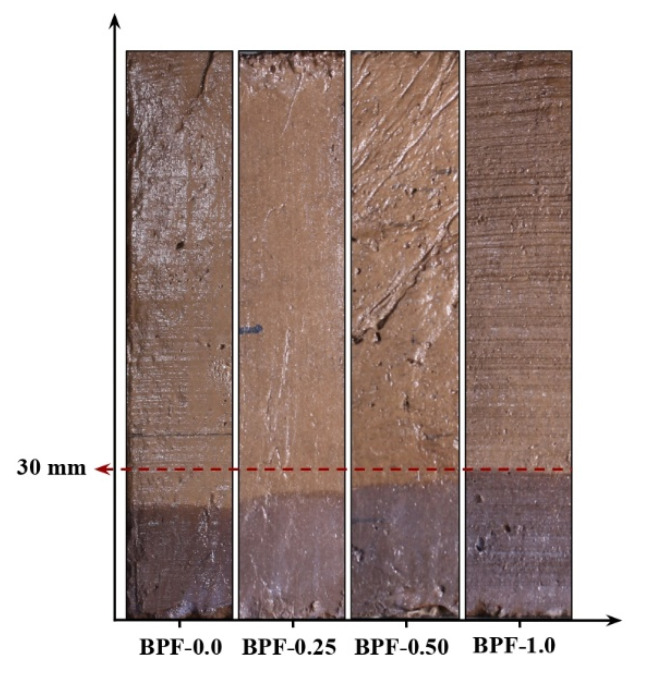
Representative capillary water rise of each earthen mix at Day 1.

Figure 7 presents the capillary water rise of each earthen mix measured at Day 1, and it can be seen that the capillary water rise monotonically increased as the BPF doses increased (e.g., at Day 1, the average values of the capillary water rise were 20, 25, 28, and 30 mm for earthen mixes BPF-0.0, BPF-0.25, BPF-0.50, and BPF-1.0, respectively).

**Figure 8:**
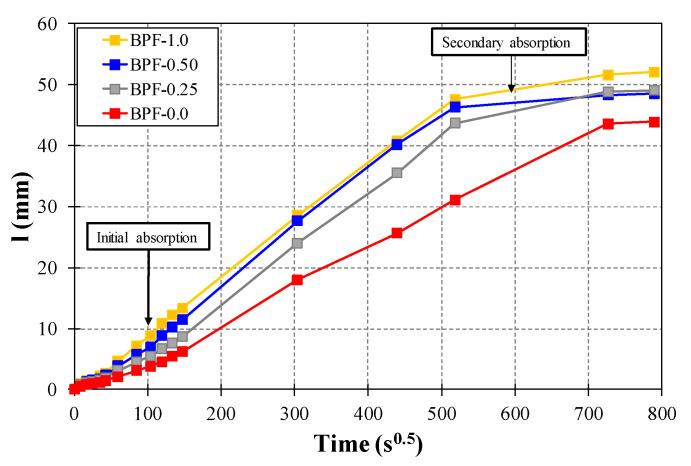
Representative curves of the capillary water absorption rate for each earthen mix.

Figure 8 presents representative curves of the capillary water absorption rate (*I*) in quantitative terms for each earthen mix. Values of *I* increased rapidly at early ages (defined as initial absorption in Figure 8) and then tended to stabilize or even reach constant values at late ages (defined as secondary absorption in Figure 8) for each earthen mix. The latter is a typical behavior related to capillary water absorption, as expressed in the standard ASTM C1585-13 [50]. Moreover, there was a monotonic increment of *I* as the BPF doses increased at each measured time. For instance, at 17.3 s^0.5^ (5 min, corresponding to the initial absorption range), the average values of *I* were 0.84, 1.26, 1.31, and 1.78 mm for earthen mixes BPF-0.0, BPF-0.25, BPF-0.50, and BPF-1.0, respectively. On the other hand, at 509 s^0.5^ (3 days, corresponding to the secondary absorption range), the average values of *I* were 33.4, 44.3, 47.9, and 48.2 mm for earthen mixes BPF-0.0, BPF-0.25, BPF-0.50, and BPF-1.0, respectively.

The monotonic increment of *I* values as the BPF doses increased can be explained considering three aspects: (i) The inclusion of BPFs modified the microstructure of the earthen matrix by increasing the number and volume of pores compared to the plain earthen mix, possibly due to fiber clusters and/or gaps between fibers and the earthen matrix [34], and this additional porosity increased the capillary water absorption through a more porous structure and gaps generated between some parts of the BPFs and the clayey matrix; (ii) BPFs have an inner hollow structure through which capillary water absorption can increase; and (iii) the chemical composition of BPFs is mainly formed of keratin, which is able to absorb and retain water rapidly, as mentioned by Bomou Ma et al. [67], further facilitating capillary water absorption.

**Figure 9:**
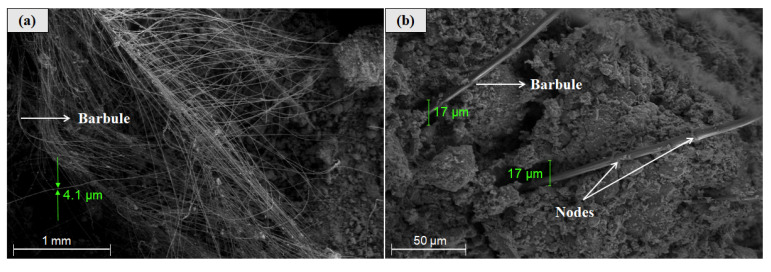
SEM of BPF-reinforced earthen mixes: (**a**) Group of barbules within the earthen matrix and (**b**) interface zone between barbules and the clayey matrix.

To further investigate the reasons behind these increments in *I* generated by the incorporation of BPFs, this study performed SEM analyses of the BPF-reinforced earthen mixes. Figure 9a shows a large group (cluster) of flexible and small barbules (4.1 µm in diameter) pulled out of the clayey matrix, and these numerous barbules provide super absorbent paths through which water can ascend within the mixes. Additionally, Figure 9b shows the interface zone between barbules and the clayey matrix, and it can be observed that the inclusion of these BPFs generated some discontinuity zones (gaps) with a micro porosity of nearly 17 µm. These incorporated micro pores modified the microstructure of the composite material, facilitating the water rise due to capillarity, as observed in this study.

**Figure 10:**
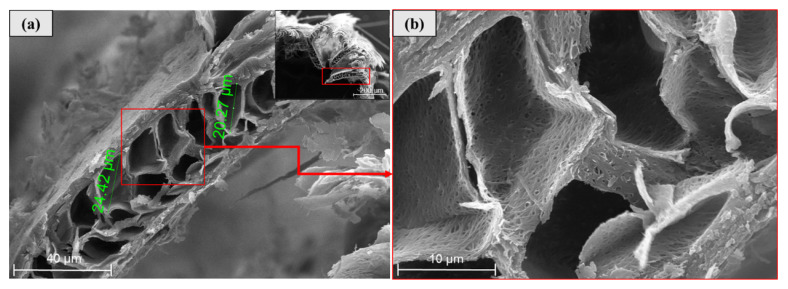
SEM of BPFs: (**a**) Barb cross section and (**b**) magnified view of the barb cross section.

Moreover, Figure 10a,b shows the cross section of a barb, which has a hollow, honeycomb-shaped inner structure with dimensions ranging from 20.27 to 24.42 µm, similar to what was obtained by Reddy et al. [41]. As mentioned before, this inner structure facilitates a capillary water increase in fiber-reinforced earthen mixes.

In statistical terms, the pair-wise ANOVA tests presented in Table 4 show that only the BPF-1.0 mix exhibited an average *I* value that was statistically significantly higher than the average *I* value corresponding to the plain earthen mix. The latter result points out that although the incorporation of BPFs facilitated capillary water absorption in earthen mixes, there was a BPF dose threshold of between 0.5% and 1.0% at which the average values of *I* were statistically affected. It is worth noting that the measurements of *I*, as well as the ANOVA tests, were performed at several different times, with each demonstrating similar trends to those described in detail in this paper, but for brevity, only the results of the ANOVA test implemented at 17.3 s^0.5^ (5 min) are presented in Table 4.

**Figure 11:**
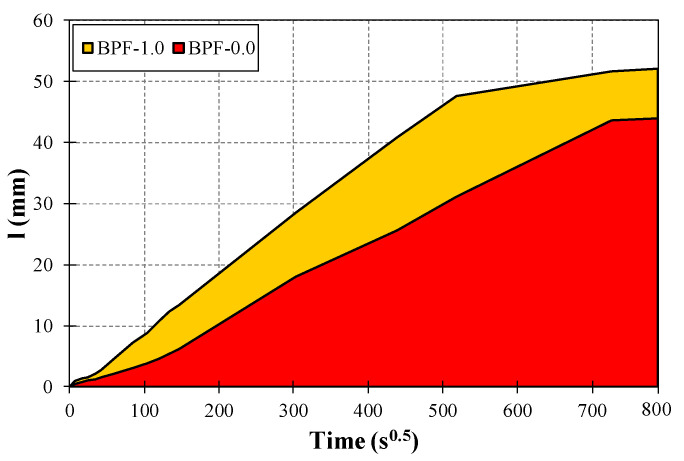
Representative areas under the capillary water absorption rate curves for BPF-1.0 and BPF-0.0 mixes.

To further describe the difference in capillary water absorption between earthen mixes BPF-0.0 and BPF-1.0, Figure 11 shows the area under the water absorption rate curves of these two earthen mixes. The resulting area for earthen mix BPF-1.0 is 39% larger than the corresponding area obtained for earthen mix BPF-0.0. This significant difference can be further related to the different capillary performances resulting from the addition of BPF in high doses (e.g., 1%). It is important to mention that the larger capillary water rise exhibited by earthen mix BPF-1.0, when compared to the remaining earthen mixes, might affect the durability of the construction materials made with this mix when exposed to water.

As previously mentioned, increments in the capillary water absorption rate might be a consequence of increments in the porosity. Table 5 presents the bulk density results, as well as the corresponding porosity estimations for each earthen mix. A monotonic increment in porosity occurred as the BPF dose increased. Even though these increments might not seem very large, there is a statistically significant difference between the average bulk density results of BPF-0.0 and BPF-1.0 mixes and their corresponding average porosity values (obtained *p*-value = 7 × 10^−5^ < level of significance = 5% for a pair-wise ANOVA test between the BPF-0.0 mix and BPF-1.0 mix). The latter is consistent with the statistically significant increment in the capillary water absorption rate value of the BPF-1.0 mix, with respect to the BPF-0.0 mix, reported in Table 4. Therefore, the incorporation of a high BFP dose (e.g., 1%) affects the microstructure of the composite material, significantly increasing the volume of voids, possibly due to the hollow inner structure of these BPFs, the formation of fiber clusters, and/or the generation of gaps between some parts of the BPFs and the matrix.

### 3.2. Compressive and Flexural Strength

**Figure 12:**
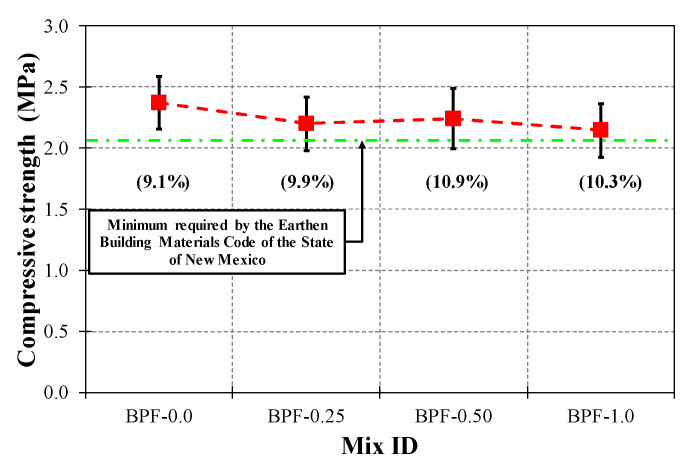
Compressive strength results for each earthen mix.

As can be seen in Figure 12, the values of AV (as squares), SD (as error bars above and below each average value), and COV (in parentheses below each average value) of the compressive strength ranged from 2.14 MPa (BPF-1.0) to 2.37 MPa (BPF-0.0), from 0.22 MPa (BPF-0.0) to 0.25 MPa (BPF-0.50), and from 9.1% (BPF-0.0) to 10.9% (BPF-0.50), respectively. It is important to note that all earthen mixes in this study exhibited average compressive strengths greater than 2.07 MPa, which is the minimum compressive strength required by the Earthen Building Materials Code of the State of New Mexico [68]. In terms of trends, there was some reduction in the average compressive strength as the BPF doses were increased. However, the latter average compressive strength reduction was not statistically significant, as confirmed by the ANOVA test summarized in Table 6, including a large *p*-value of 0.11, which is consistent with previous works addressing the compressive strengths of earthen mixes incorporating small doses of polypropylene micro fibers [48,59].

**Figure 13:**
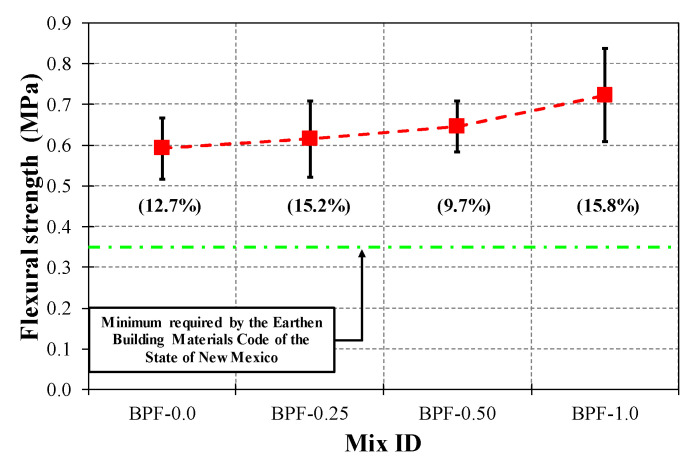
Flexural strength results for each earthen mix.

In terms of the flexural performance, Figure 13 shows that the AV, SD, and COV values of flexural strength ranged from 0.59 MPa (BPF-0.0) to 0.72 MPa (BPF-1.0), from 0.06 MPa (BPF0.50) to 0.11 MPa (BPF-1.0), and from 9.7% (BPF-0.50) to 15.8% (BPF-1.0), respectively. It is worth noting that all earthen mixes in this study exhibited average flexural strengths greater than the minimum value of 0.35 MPa required by The Earthen Building Materials Code of the State of New Mexico [68]. In terms of trends, there was a monotonic increment in the flexural strength as the dose of BPFs increased. Nevertheless, similar to what was observed with compressive strengths, the latter monotonic flexural strength increment was not statistically significant, as confirmed by the ANOVA test summarized in Table 7. The latter is also consistent with previous works addressing the flexural strengths of earthen mixes incorporating small doses of polypropylene micro fibers [48,59], where these micro fibers, when well mixed and in reduced doses, did not significantly affect the mechanical strength properties.

It is important to mention that the negligible effect of the incorporation of BPFs on the compressive and flexural strength results of the earthen mixes obtained in this study is consistent with the results obtained by Araya-Letelier et al. [35]. Moreover, the compressive and flexural strength results obtained by this study were, overall, very similar to the values obtained by Araya-Letelier et al. [35] (e.g., the average compressive strength results for earthen mix BPF-1.0 were 2.14 MPa in this study and 2.07 MPa in the study by Araya-Letelier et al. [35]). The latter can be used to validate a proper manual manufacturing process of the new samples of this study. Additionally, this study used smaller specimens to evaluate both compressive and flexural strengths (e.g., this study used 160 × 40 × 40 mm RILEM beams to evaluate flexural strengths, whereas the previous study [35] used 310 × 105 × 70 mm beams) and some small strength increments were exhibited in this study with respect to the previous study [35]. The latter might be explained by Weibull’s size effect theory, which states that with an increase of the geometric size of specimens, the probability of reaching a lower strength increases [69]. Moreover, it is also worth mentioning that, as expected, the incorporation of BPFs had minor to null effects on the mechanical strengths because micro fibers mostly provide crack control due to the tensile stress transfer capability of the fibers across cracked surfaces, which is known as crack-bridging. In this way, micro fibers provide significant shear resistance across developing cracks that leads to an enhanced post-cracking performance in terms of energy dissipation, the pseudo-ductile tensile response, and toughness, relative to the brittle behavior of plain earthen mixes such as concrete [70,71]. For example, in terms of flexural toughness, the previous study by Araya-Letelier et al. [35] addressing the effect of BPFs on earthen mixes found that the flexural toughness of BPF-reinforced earthen mixes was up to 134% greater than the value corresponding to unreinforced (plain) mixes, which is consistent with the significant impact of micro fibers on the post-cracking performance rather than on the mechanical strength.

### 3.3. Impact Strength

**Figure 14:**
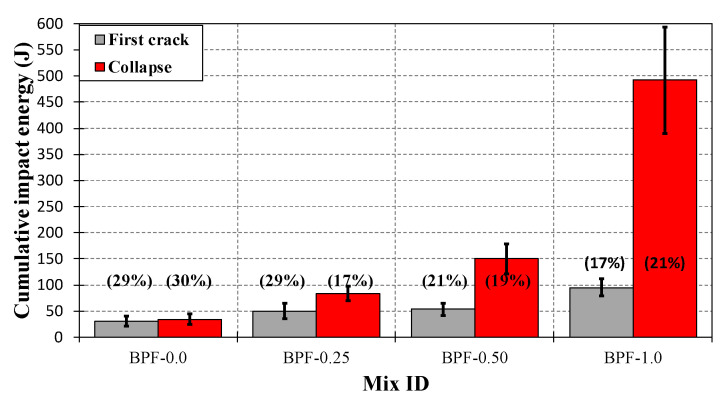
Impact strength for each earthen mix.

As can be seen in Figure 14, the values of AV (as bars), SD (as error bars above and below each average value), and COV (in parentheses) of the first crack energy ranged from 30.9 J (BPF-0.0) to 95.2 J (BPF-1.0), from 9.09 J (BPF-0.0) to 16.3 J (BPF-1.0), and from 17% (BPF-1.0) to 29% (BPF-0.0), respectively. Moreover, Figure 14 also shows that the values of AV, SD, and COV of collapse energy ranged from 34.1 J (BPF-0.0) to 492.1 J (BPF-1.0), from 10.1 J (BPF-0.0) to 102.0 J (BPF-1.0), and from 30% (BPF-0.0) to 17% (BPF-0.25), respectively. From the latter values, it can be observed that the incorporation of increasing doses of BPFs in earthen mixes monotonically increased the impact strength in terms of both cumulative energy at first crack (e.g., by three times for BPF-1.0 with respect to BPF-0.0) and cumulative energy at collapse (e.g., by 14 times for BPF-1.0 with respect to BPF-0.0). The latter behavior is consistent with previous studies addressing the effect of other fibers on the impact performance of earthen mixes [11,35,50].

To further support the previous analyses, Table 8 and Table 9 provide the results of the ANOVA tests for the impact strength at first crack and at collapse, respectively. It can be observed that all the BPF-reinforced earthen mixes exhibit statistically significant larger values of average impact strength for both the first crack and collapse performance, compared to BPF-0.0, which can be additionally explained by the very small resulting *p*-values. These results suggest that the fracture toughness performance of BPF-reinforced earthen mixes is greatly improved compared to plain earthen mixes due to adequate bonding between BPFs and the matrix, as this facilitates stress transfer to the BPFs when the clayey matrix is damaged.

### 3.4. Dry Abrasion Resistance

**Figure 15:**
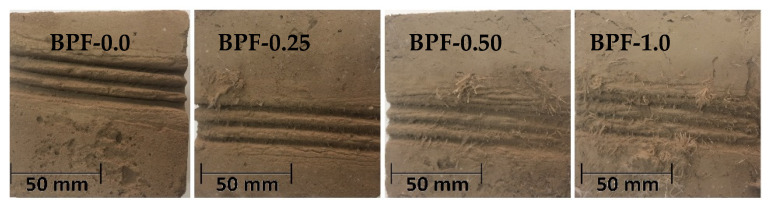
Representative images of the dry abrasion resistance of each earthen mix.

Figure 15 presents representative images of the dry abrasion resistance of each earthen mix assessed in this study. It can be observed that increasing doses of BPFs monotonically increased the dry abrasion resistance of earthen mixes and, consequently, also enhanced the durability of these mixes.

**Figure 16:**
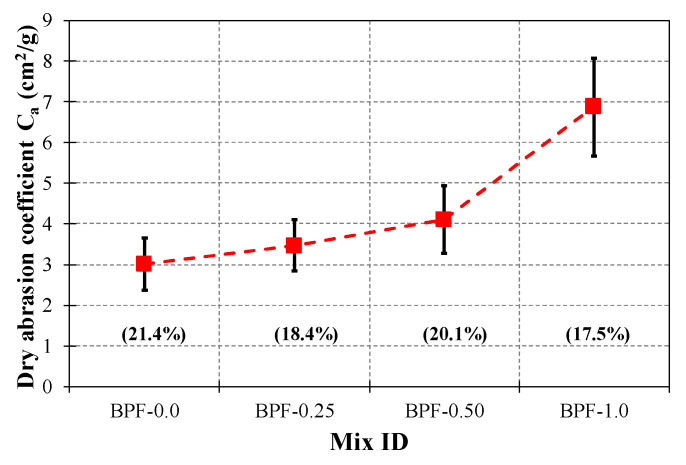
Dry abrasion coefficient results of each earthen mix.

Quantitatively, Figure 16 shows that the AV, SD, and COV values of the dry abrasion coefficient (C_a_) ranged from 3.0 cm^2^/g (BPF-0.0) to 6.9 cm^2^/g (BPF-1.0), from 0.64 cm^2^/g (BPF-0.0) to 1.2 cm^2^/g (BPF-1.0), and from 17.5% (BPF-1.0) to 21.4% (BPF-0.0), respectively. As shown by Figure 16, the best dry abrasion resistance performance was obtained by BPF-1.0, whose average C_a_ value was 2.3 times greater than the average C_a_ value of the plain earthen mix. The improved behavior of BPF-reinforced earthen mixes, compared to BPF-0.0, can be explained by the relatively large aspect ratio of the fibers, as well as the rough external surface (due to the presence of barbules), which provides adequate bonding between BPFs and the clayey matrix. However, more work addressing the interface zone between BPFs and the clayey matrix (e.g., a pull-out test) is recommended to better understand this bonding.

The ANOVA test results of C_a_ are presented in Table 10, which shows a statistically significant improved average dry abrasion resistance behavior of earthen mixes BPF-0.5 and BPF-1.0 compared to BPF-0.0. However, earthen mix BPF-0.25 did not demonstrate a statistically significant improved average performance compared to BPF-0.0, as further explained by the large *p*-value of 0.23 for the pair-wise test between earthen mixes BPF-0.0 and BPF-0.25. It is possible that the smaller BPF dose used in BPF-0.25 (compared to earthen mixes BPF-0.50 and BPF-1.0) did not provide enough fibers to bond with the damaged clayey matrix and maintain the integrity of the material.

## 4. Conclusions and Comments

This study assessed the waste valorization of chicken feathers (CFs) from the poultry industry as a sustainable biopolymer fiber (BPF) reinforcement alternative for earthen mixes intended for earthen construction, such as adobe masonry, rammed earth, and earthen plasters and mortars, among others. In particular, four earthen mixes with increasing doses of BPFs (i.e., 0%, 0.25%, 0.5%, and 1% of BPFs by weight of soil) were developed to evaluate the effects of BPF-reinforcement on their capillary, mechanical, impact, and abrasion performance. The following conclusions can be drawn:

1. The capillarity was monotonically increased by the incorporation of BPFs, but this increment was only statistically significant for the BPF-1.0 mix with respect to BPF-0.0. The latter can be explained since the incorporation of BPFs monotonically increased the porosity of the mixes for increasing doses of BPFs (possibly due to the hollow inner structure and keratin nature of the BPFs, the formation of fiber clusters, and/or the generation of small gaps between some parts of the interface between BPFs and the clayey matrix), which facilitated the capillary water rise;
2. Even though some variations were observed, the mechanical performance, measured in terms of the compressive and flexural strength, was not statistically modified. This is consistent with previous studies addressing the incorporation of polypropylene micro fibers into earthen mixes, where these micro fibers, when well mixed and in reduced doses, did not significantly affect the mechanical properties. However, as reported in a previous study, the incorporation of BPFs has a positive effect on the post-cracking performance of earthen mixes, especially in terms of the flexural toughness;
3. Regarding the impact strength, increasing doses of BPFs monotonically increased both the impact energy at the first crack (e.g., by three times for BPF-1.0 with respect to BPF-0.0) as well as the impact energy at collapse (e.g., by 14 times for BPF-1.0 with respect to BPF-0.0), suggesting that an improved fracture toughness performance can be expected when adding these waste-based fibers. The improvements in impact strength were statistically significant, even for small doses of BPFs (e.g., 0.25%);
4. BPFs increased the abrasion resistance of earthen mixes by up to 130% compared to BPF-0.0, but this increase was only statistically significant for BPF-0.5 and BPF-1.0. The latter reflects the need for a minimum dose of BPF to bond with the clayey matrix and significantly impact the abrasion resistance.

Although there are some challenges related to water absorption and the mixing of BPFs (and natural fibers in general), this paper recommends incorporating these waste-based BPFs into earthen mixes, especially at a 1% dose. Although the capillary water absorption performance was inferior with the incorporation of high doses of BPFs (e.g., 1%), the impact strength and abrasion resistance were enhanced without statistically affecting either the compressive or flexural strength.

## Figures and Tables

**Table 1:** Main morphological, physical, and mechanical characteristics of biopolymer fibers (BPFs) ^1^.

Diameter (mm)	Length (mm)	Aspect Ratio (Length/Diameter)	Density ^2^ (g/cm ^3^)	Water Absorption (%)	Elongation ^2^ (%)	Tensile Strength ^2^ (MPa)	Young’s Modulus ^3^ (MPa)
0.012–0.11	0.3–12	147–354	0.8–0.89	85	7.7 ± 0.85	187.2 ± 0.46	3590 ± 1090

^1^ Ranges are provided when available and single numbers report average values; ^2^ from [41]; ^3^ from [45].

**Table 2:** Earthen mix ID codes and proportions of materials used in each mix.

Mixes ID Code	Clayey Soil ^1^ (kg)	Water (kg)	W/S (%)	BPFs ^1^ (kg)	BPFs ^2^ (%)
BPF-0.0	100	25	25	0.0	0.0
BPF-0.25				0.25	0.25
BPF-0.50				0.50	0.50
BPF-1.0				1.0	1.0

^1^ Oven-dry condition; ^2^ weight of oven-dry BPFs to weight of oven-dry soil.

**Table 3:** Specimens prepared in this study.

Specimens	Dimensions (mm)	Experimental Methods	
		Type	Specimens Per Type of Test for Each Earthen Mix
Prismatic	155 × 105 × 70	Capillarity	4
RILEM beam	160 × 40 × 40	Flexural and compressive strength	6 (flexural strength)/ 12 (compressive strength)
Beam	310 × 105 × 70	Impact strength	6
Cube	100 × 100 × 100	Dry abrasion resistance	6

**Table 4:** 5% significance level ANOVA tests for the capillary water absorption rate (*I*) of earthen mixes at 17.3 s^0.5^ (300 s).

ANOVA Test	Source	DF	SS	MS	f_st_	F_cr_	*p*-Value	Statistically Significant?
All earthen mixes (one way)	Treatment (BPF)	3	1.777	0.592	3.768	3.49	0.0408	Yes
	Error	12	1.886	0.157				
BPF-0.0 versus BPF-0.25 (pair-wise)	Treatment (BPF)	1	0.349	0.349	3.093	5.99	0.0129	No
	Error	6	0.676	0.113				
BPF-0.0 versus BPF-0.50 (pair-wise)	Treatment (BPF)	1	0.427	0.427	3.469	5.99	0.111	No
	Error	6	0.738	0.123				
BPF-0.0 versus BPF-1.0 (pair-wise)	Treatment (BPF)	1	1.769	1.769	18.98	5.99	0.005	Yes
	Error	6	0.559	0.093				

**Table 5:** Porosity values for each earthen mix.

Earthen Mix	Bulk Density ^1^ (kg/m^3^)	Porosity (%)
BPF-0.0	1739.2 {1.7%}	29.6
BPF-0.25	1705.1 {2.3%}	30.9
BPF-0.50	1672.7 {2.3%}	32.3
BPF-1.0	1622.4 {1.8%}	34.3

^1^ COV values in curly brackets.

**Table 6:** 5% significance level ANOVA tests for the compressive strength of earthen mixes at 28 days.

ANOVA Test	Source	DF	SS	MS	f_st_	F_cr_	*p*-Value	Statistically Significant?
All earthen mixes (one way)	Treatment (BPF)	3	0.327	0.1091	2.14	2.84	0.1076	No
	Error	44	2.234	0.0508				

**Table 7:** 5% significance level ANOVA tests for the flexural strength of earthen mixes.

ANOVA Test	Source	DF	SS	MS	f_st_	F_cr_	*p*-Value	Statistically Significant?
All earthen mixes (one way)	Treatment (BPF)	3	0.059	0.0197	2.50	3.10	0.089	No
	Error	20	0.157	0.0079				

**Table 8:** 5% significance level ANOVA tests for the impact strength at the first crack of earthen mixes.

ANOVA Test	Source	DF	SS	MS	f_st_	F_cr_	*p*-Value	Statistically Significant?
All earthen mixes (one way)	Treatment (BPF)	3	1.3 × 10^4^	4.4 × 10^3^	25.49	3.10	<1 × 10^−5^	Yes
	Error	20	3.4 × 10^3^	1.7 × 10^2^				
BPF-0.0 versus BPF-0.25 (pair-wise)	Treatment (BPF)	1	1.1 × 10^3^	1.1 × 10^3^	7.43	4.96	0.0214	Yes
	Error	10	1.5 × 10^3^	1.5 × 10^2^				
BPF-0.0 versus BPF-0.50 (pair-wise)	Treatment (BPF)	1	1.6 × 10^3^	1.6 × 10^3^	14.51	4.96	0.0034	Yes
	Error	10	1.1 × 10^3^	1.1 × 10^2^				
BPF-0.0 versus BPF-1.0 (pair-wise)	Treatment (BPF)	1	1.2 × 10^4^	1.2 × 10^4^	71.29	4.96	<1 × 10^−5^	Yes
	Error	10	1.7 × 10^3^	1.7 × 10^2^				

**Table 9:** 5% significance level ANOVA tests for the impact strength at the collapse of earthen mixes.

ANOVA Test	Source	DF	SS	MS	f_st_	F_cr_	*P*-Value	Statistically Significant?
All earthen mixes (one way)	Treatment (BPF)	3	7.7 × 10^5^	2.6 × 10^5^	89.29	3.10	<1 × 10^−5^	Yes
	Error	20	5.8 × 10^4^	2.9 × 10^3^				
BPF0.0 versus BPF0.25 (pair-wise)	Treatment (BPF)	1	7.4 × 10^3^	7.4 × 10^3^	50.03	4.96	3 × 10^−5^	Yes
	Error	10	1.5 × 10^3^	1.5 × 10^2^				
BPF0.0 versus BPF0.5 (pair-wise)	Treatment (BPF)	1	4.0 × 10^4^	4.0 × 10^4^	88.88	4.96	<1 × 10^−5^	Yes
	Error	10	4.5 × 10^3^	4.4 × 10^2^				
BPF0.0 versus BPF1.0 (pair-wise)	Treatment (BPF)	1	6.3 × 10^5^	6.3 × 10^5^	119.8	4.96	<1 × 10^−5^	Yes
	Error	10	5.3 × 10^4^	5.3 × 10^3^				

**Table 10:** 5% significance level ANOVA tests for the dry abrasion coefficient of earthen mixes.

ANOVA Test	Source	DF	SS	MS	f_st_	F_cr_	*p*-Value	Statistically Significant?
All earthen mixes (one way)	Treatment (BPF)	3	5.4 × 10^1^	1.8 × 10^1^	24.38	3.10	<1 × 10^−5^	Yes
	Error	20	1.5 × 10^1^	0.7 × 10^0^				
BPF-0.0 versus BPF-0.25 (pair-wise)	Treatment (BPF)	1	0.7 × 10^0^	0.7 × 10^0^	1.61	4.96	0.2335	No
	Error	10	4.1 × 10^1^	0.4 × 10^0^				
BPF-0.0 versus BPF-0.50 (pair-wise)	Treatment (BPF)	1	3.6 × 10^0^	3.6 × 10^0^	6.68	4.96	0.0272	Yes
	Error	10	5.5 × 10^0^	0.6 × 10^0^				
BPF-0.0 versus BPF-1.0 (pair-wise)	Treatment (BPF)	1	4.5 × 10^1^	4.5 × 10^1^	48.14	4.96	4 × 10^−5^	Yes
	Error	10	9.3 × 10^0^	0.9 × 10^0^				

## Abbreviations

The following abbreviations are used in this manuscript:

AV	Average
BPF	Biopolymer fiber
BPF-0.0	Earthen mix incorporating 0% of BPFs (by weight of clayey soil)
BPF-0.25	Earthen mix incorporating 0.25% of BPFs (by weight of clayey soil)
BPF-0.50	Earthen mix incorporating 0.50% of BPFs (by weight of clayey soil)
BPF-1.0	Earthen mix incorporating 1.0% of BPFs (by weight of clayey soil)
CF	Chicken feather
CL	Low plasticity clay
COV	Coefficient of variation
EDS	Energy dispersive X-ray spectroscopy
F_crit_	Value of an F-distribution that captures a specific area under the right tail
f_st_	Ratio between mean square (variance) between study groups and mean square (variance) within each study group
ID	Identification code
SD	Standard deviation
SEM	Scanning electron microscopy

## Notation List

The following notation list defines the meaning of each symbol used in Equations (1)–(3) and throughout the text.

I	Water absorption rate
∆m	Mass variation between two consecutive measurements
a	Cross section exposed to water absorption
d	Water density
P	Porosity
D	Bulk density
$W_{s}$	Specific weight of solids
$C_{a}$	Dry abrasion coefficient
S	Brushed area
$m_{0}$	Mass before brushing
$m_{1}$	Mass after brushing
